# An Improved Design of the Spiral-Coil EMAT for Enhancing the Signal Amplitude

**DOI:** 10.3390/s17051106

**Published:** 2017-05-12

**Authors:** Xiaojuan Jia, Qi Ouyang, Xinglan Zhang

**Affiliations:** Institute of Smart Engineering, School of Automation, Chongqing University, Chongqing 400044, China; jiaxiaojuan@cqu.edu.cn (X.J.); zxlan@cqut.edu.cn (X.Z.)

**Keywords:** electromagnetic ultrasonic transducer, double-coil, finite element model, signal-to-noise ratio, bulk wave

## Abstract

The low energy transition efficiency of electromagnetic ultrasonic transducer (EMAT) is a common problem in practical application. For the purpose of enhancing the amplitude of the received signal, an improved double-coil bulk wave EMAT is proposed for the thickness measurement of metallic block. This new configuration of magnets consists of a solid cylindrical magnet and a ring-shaped magnet encircling the outer side of the solid cylindrical one. A double-coil was applied instead of a single spiral-coil. Numerical simulations were performed to analyze and optimize the proposed configuration of the EMAT by the 2-D axisymmetric finite element model (FEM). The experiment effectively verifies the rationality of the new configuration and the feasibility of improving the signal strength.

## 1. Introduction

Nondestructive testing (NDT) is an indispensable tool for industrial development, and its method mainly includes ultrasonic testing (UT), radiation testing (RT), magnetic particle testing (MT), liquid penetration testing (PT) and AC field measurement (ACFMT). Electromagnetic acoustic transducer (EMAT) is a type of non-contact ultrasonic transducer, which is the core component of ultrasonic excitation and acceptance in UT [1,2,3,4,5]. Due to a plenty of outstanding advantages such as non-contact operation, requiring no coupling fluid, the flexibility to generate and detect multiple wave modes, and the capability of operating in high temperature, isolation layer and other harsh environment, EMATs are particularly useful and active in nondestructive evaluation (NDE) and internal structure evaluation (ISE) [6,7,8,9,10,11,12]. Besides, EMATs have be distinctively attractive in thickness measurement for their good penetration ability.

However, compared with conventional piezoelectric transducers, the poor energy efficiency is the predominant drawback of EMATs, which leads to the low signal to noise ratio (SNR) of the ultrasonic signal [13,14,15,16]. This drawback severely limits the application of EMATs in various fields. So it is urgently needed to make EMATs have a higher SNR and extraordinary pure ultrasound with a reasonable design. Earlier research was made based on the experimental approach, which was costly and time-consuming. Until recently, researchers established the mathematical model of EMATs, and made remarkable improvement in theoretical analysis and numerical simulation [17]. Now, there are substantial conspicuous improvements in the performance and transduction efficiency of EMATs, such as the application of high-frequency pulse generator and low-noise receiver, the introduction of phased array and the digital signal processing [18,19]. However, these methods will make electronic devices bulky. Under the high voltage environment, there are hidden dangers. For example, the static electricity is easily to result fire in gas and oil industries [20].

Obviously, it can be obtained from Equation (1) that the square of the magnetic flux density is proportional to the SNR of EMATs with the mechanism of Lorentz force [7,8,9,10,11,12,13,14,15]. The SNR can be given as

(1) $$\frac{V_{\mathrm{EMAT}}}{V_{\mathrm{noise}}}=\frac{\sqrt{P_{0}}N^{2}B^{2}A}{2Z_{S}\sqrt{KT\beta }R_{\mathrm{EMAT}}}\exp\left(-\frac{\alpha h}{D}\right)$$

where $V_{\mathrm{EMAT}}$ is the received voltage across the terminals of the coil (V), $V_{\mathrm{noise}}$ is the received noise level (V), $P_{0}$ is the power of the EMAT (W), N is the number of turns per unit length, B is the magnetic flux density (T), A is the active area of EMAT ($m^{2}$), $Z_{S}$ is the acoustic impedance of the part (Ω), either longitudinal or shear wave, K is Boltzmann’s constant times electronic charge (Meter-Kilogram-Second units), T is temperature in degrees Kelvin (K), β is the bandwidth of the amplifier (Hz), $R_{\mathrm{EMAT}}$ is the resistance of EMAT coil (Ω), α is a geometry dependent constant, h is the lift-off distance between EMAT coil and the surface of the part (m), and D is the diameter of the coil (m). Therefore, it is an extremely effective way to improve the transducer efficiency by enhancing the magnetic flux density. Consequently, improvement in the strength of the magnetic field based on simulation and experiment methods has been widely applied to ameliorate SNR of the EMAT. Pei et al. enhanced the efficiency of meander-line-coil (MLC) EMAT by improving the magnetic field [7]. Wang et al. increased the excitation efficiency of a torsional wave PPM EMAT array for pipe inspection by optimizing the element number of the array based on 3-D FEM [8]. Kang et al. improved the signal amplitude of surface wave EMAT based on 3-D simulation analysis and orthogonal test method [9]. Xie et al. proposed a wholly analytical modelling method to study the Rayleigh waves’ beam directivity on the surface of the material [10]. Seung et al. presented a new EMAT for generation and measurement of omnidirectional shear-horizontal (SH) guided waves in metallic plates [11]. There have been previous studies of SH wave EMAT, but they mainly focused on the meander-line-coil (MLC) EMAT for surface wave generation and detection in the metallic plate or pipe [7,8,9,10].

Until now, there is hardly research about the bulk wave EMAT, which is composed of spiral-coil and cylindrical magnet, except that Julio Isla et al. presented an in-depth analysis of the bias magnetic field strength and resulted signal amplitude of different magnet configurations for shear wave EMAT on mild steel [20]. Nevertheless, in practice, the thickness measurement has a wide range of applications by bulk waves EMAT. And a higher signal amplitude can help to improve the penetration depth and the measurement accuracy of the EMAT system. Accordingly, it is of great practical significance to improve the energy efficiency of bulk wave EMAT for thickness measuring of the metallic material.

**Figure 1:**
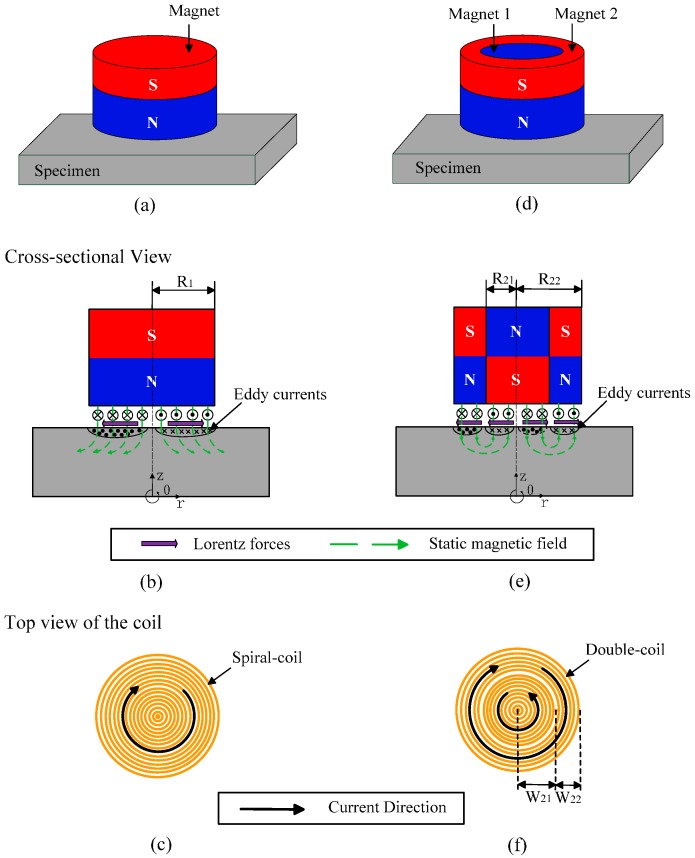
Schematics of the conventional spiral-coil EMAT and the improved double-coil EMAT.(**a**,**d**) 3-D model; (**b**,**e**) cross-sectional view; (**c**,**f**) top view of the coil of the two configurations, respectively.

The main work of this paper is to propose an improved configuration related to cylindrical magnet to strengthen the magnetic field of the spiral-coil EMAT for generation and detection of the bulk waves, which is mainly used to measure the thickness of the metallic plate. It has been reported that the signal intensity of the bulk wave is increased by changing the configuration of a magnet with a racetrack coil, but not referenced to the spiral-coil [21]. Figure 1 shows a comparison of the diagrammatic sketch of the modified magnet arrangement with the conventional one. The detailed structure of the proposed EMAT will be described in Section 2. Then, by using the axisymmetric finite element model (FEM), the distribution of the magnetic flux density with respect to the traditional and improved double-coil EMAT is analyzed by numerical simulation. Subsequently, in Section 3, the radius ratio and height of the new EMAT were optimized to find the most suitable configuration when other dimensions are constant. In the last section of the paper (Section 4), we conclude that the proposed magnet configuration can tremendously improve the signal amplitude compared with the traditional configuration by numerical simulation and experimental verification.

## 2. Configuration of the Double-Coil EMAT and Numerical Simulation

In this section, the modified configuration of the bulk-wave EMAT configuration will be described in detail, and it will be compared with the traditional configuration. In order to verify the rationality of the new configuration, a two dimensional axisymmetric finite element model (2-D FEM) is established due to its axial symmetry. The numerical simulation and calculation indicate that the improved configuration dramatically heighten the magnetic flux density.

### 2.1. Improved Double-Coil EMAT Design

It is well known that the combination of the cylindrical magnet providing static bias magnetic field perpendicular to the surfaces of the tested sample and the spiral coil exerting high-frequency pulse excitation produces ultrasonic guided waves within the specimen [22]. As shown in Figure 1a, the spiral-coil EMAT can generate bulk waves in samples for thickness measurement and internal defect detection of a metallic plate. But the conversion efficiency is not ideal in practice. In order to overcome this conundrum, an improved magnet arrangement is proposed, which consists of a solid cylindrical magnet (Magnet 1) and a hollow annular magnet (Magnet 2) with axial polarization. And the polarization direction of the two magnets is placed oppositely, as shown in Figure 1b,e. It is important to notice that the solid cylinder is tightly surrounded by the ring-shaped permanent magnet, as shown in Figure 1d. In other words, the outer diameter of the solid cylindrical magnet is equal to the inner diameter of the annular hollow magnet. 

To make the direction of the Lorentz force all outward along the radial direction, a double-coil is used instead of a single spiral-coil, as shown in Figure 1f. The double-coil includes two coils that are interconnected to each other. The width of the inner coil is $W_{21}$ which is equal to the radii of the Magnet 1. And the width of the outer coil is $W_{22}$, which is equal to the difference between the radii of the Magnet 1 ($R_{21}$) and Magnet 2 ($R_{22}$). The current flow direction in the coil located below the cylindrical magnet (Magnet 1) is opposite to that of the coil under the hollow circular magnet (Magnet 2). Note that all the magnets (Neodymium Iron Boron) have the same remanence. An aluminum plate with thickness of 50 mm is taken as a specimen.

Generally speaking, when two magnets are close to each other, like magnetic poles repel each other, whereas opposite poles attract each other. Therefore, the magnetic flux density of the conventional EMAT is scattered around the magnet due to reversal placement of the magnetic pole and far distance between two poles. Differently, the magnetic flux density will focus on the surface of the magnet in the case of the improved EMAT configuration, as shown in Figure 1e. This analogical configuration combined with a wound coil has been used to generate omnidirectional SH waves, but not yet combined with the spiral-coil to generate bulk waves [11].

### 2.2. Numerical Simulation of the Static Magnetic Field

In this section, the distribution of the magnetic flux density with respect to the conventional and modified configuration will be compared by numerical calculation. The 2-D FEM was established with COMSOL Multiphysics [23]. A 2-D axisymmetric space dimension chosen for the coil and magnet are axisymmetric about *z* axis. The AD/DC, magnet field module were used to generate Lorentz force and the structural mechanics module was applied to produce and propagate the bulk wave. Because of a coupling of varieties of physical field, the coupling variables need to be set up. The vector product of the magnetic flux intensity ($B_{0}$) of the bias magnetic and eddy current density ($J_{e}$) set to be the volume force ($f_{L}$) in the specimen for the transmitting process. The $f_{L}$ can be given by 

(2) $$f_{L}=B_{0}\times J_{e}$$

And the vector product of the magnetic flux density ($B_{0}$), electrical conductivity of the specimen (σ) and the velocity of the charged particles (*v*) set to be the eddy current density ($J_{L}$) of the pulsed eddy current field for the receiving process. The $J_{L}$ under the surface of a conductive specimen with density 

(3) $$J_{L}=\sigma \left(v\times B_{0}\right)$$

Then, the eddy current is inductively detected by the coil of the EMAT. Because the square of the magnetic flux density is proportional to the SNR of EMAT, it contributes twice in the process of transmitting and receiving for an EMAT in pulse-echo configuration [20].

The 2-D FEM consists of the magnet, coil, aluminum plate, air and infinite element domains. The side and bottom of the aluminum plate be defined as absorbing boundary. In order to ensure the consistency, the magnet of the two configurations is loaded with the same residual magnetic flux density of 1.2 T (grade: 35 N) and axial polarization, differing only in the configuration of the magnets and the structure of the coil.

**Figure 2:**
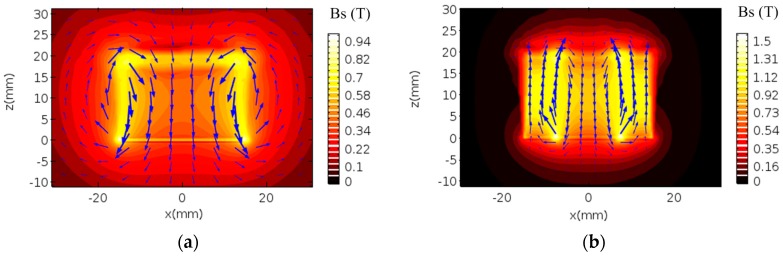
Magnetic flux density distributions in two configurations for (**a**) conventional and (**b**) improved double-coil EMAT.

To give a fair comparison, the volume of the conventional magnet is equal to that of the improved one. In the conventional configuration, the radii of the magnet is 15 mm. While, in the improved configuration, the solid magnet (Magnet 1) with radii of 10 mm and the ring magnet (Magnet 2) with inner and outer radii of 10 mm and 15 mm were applied in the 2-D FEM. And all the height of the magnet is 20 mm. The coil size used in both configurations is same with a diameter of 0.51 mm and a number of turns of 30, and the material of the coil is copper wire. All parameters of the 2-D FEM are listed in Table 1. Under the two configurations, the distribution of the magnetic flux density in the surface of the specimen was illustrated after numerical calculation, as shown in Figure 2. It should be noted that the graph is a cross section of a 2-D axisymmetric graph rotated around the *z* axis.

Figure 2 shows the distribution of the magnetic flux density in two cases. Blue arrows represent the direction of the magnetic field. It can be seen from Figure 2a, the magnetic flux density of the traditional configuration mainly scatters around the magnet, which direction is perpendicular to the surface of the sample. As the distance from the magnet is farther, the magnetic flux density decreases gradually within a certain range around the magnet. However, the magnetic flux density of the new configuration is more concentrated in the fringe and internal of the magnet, as shown in Figure 2b. In a certain range around the magnet, with the distance from magnet increasing, the magnetic flux density decreases sharply. Significantly, it can be seen from the cutline on the right side of the each photograph that the magnetic flux density of the modified configuration has a larger peak at the intersection of two magnets, which is helpful to improve the conversion efficiency.

**Figure 3:**
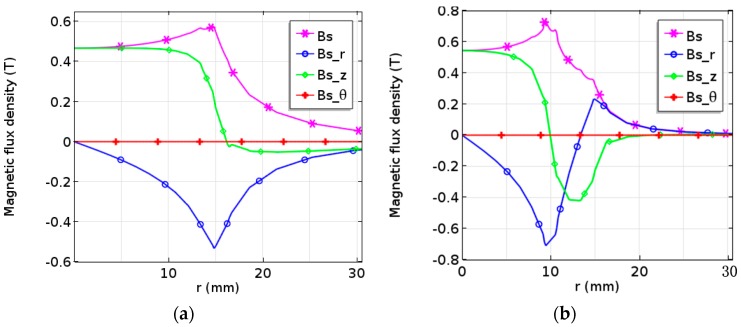
Magnetic flux density distributions of 2-D axisymmetric model for (**a**) conventional and (**b**) improved double-coil EMAT on the upper surface of the specimen.

In order to accurately analyze the magnitude of the magnetic flux density, the magnetic flux density vector in the surface of the aluminum plate was also calculated. Figure 3 shows the distribution of magnetic flux density with respect to the conventional and modified EMAT. It is important to see that the magnitude scales of Figure 3a,b are different. It can be seen from Figure 3a, the radial component of magnetic flux density ($B_{s\_r}$) is equal to 0 in the center of the magnet, and a spike peak appears near the edge of the magnet. The axial component of magnetic flux density ($B_{s\_z}$) has an arched peak on the surface of the magnet and sharply decreases to 0 or even negative near the edge of the magnet. The peak of the magnetic flux density ($B_{s}$) occurs at the edge of the magnet, with a maximum magnitude of 0.56 T. However, the distribution of the magnetic flux density that respect to the modified EMAT is different from that of the conventional one, as shown in Figure 3b. $B_{s\_r}$ and $B_{s\_z}$ have two spike peaks and arched peaks, respectively. $B_{s}$ emerges a large peak at the junction of two magnets, with a maximum amplitude of 0.78 T.

It is worth noting that, in both configurations, the circumferential magnetic flux density ($B_{s\_\theta }$) in the direction that perpendicular to the cross section maintains 0, perpetually. This is due to the polarization direction of the magnetic field in z axis. Since only the arrangement of magnets is changed, the magnetic flux density of the double-coil EMAT increases by about 40%. According to Equation (1), the magnetic flux density is proportional to the SNR. Therefore, this new configuration is advantageous for improving the conversion efficiency of the transducer.

### 2.3. Simulation of the Dynamic Characteristics

The ultrasonic signals under two configurations are compared and analyzed in this section. Generally, there are three mechanisms influencing on the ultrasonic transmission of EMAT: Lorentz force that applies to non-ferromagnetic materials, magnetostriction force and magnetization force, which are suitable for ferromagnetic materials [24]. Therefore, in aluminum, Lorentz force plays a dominant role in the numerical simulation of the transmission and reception of bulk waves. Based on Lorentz force mechanism, the working process of EMATs is that the ultrasound bulk waves propagating perpendicular to the surface of the sample is excited when the specimen is subjected to Lorentz force acting along the radial direction of the coil under the action of bias magnetic field.

In this paper, the 2-D axisymmetric FEM was established based on Lorenz force mechanism and ignored other mechanisms for an aluminum plate as a sample to be tested, which includes both the coil and the specimen transmitting ultrasound guided waves.

To facilitate the numerical analysis, the transmitting EMAT is assumed to be installed on a 200-mm-thick aluminum plate. The dimension and material properties of the magnet and coil are the same as those of the static simulation, respectively. The lift-off distance between the magnet and the upper surface of the aluminum plate is set to 0.5 mm. The coil is driven by a three-cycle-long current signal with the peak value of 200 A, which centered at 2 MHz and modulated by a sine squared window. Considering, the first step is to calculate the static bias magnetic field in the finite element simulation, which requires a certain amount of time to establish a stable magnetic field, so the excitation signal was loaded into the model at 6 μs. In order to guarantee the smoothness and stability of the results, the step length was set to 0.1 μs.

**Figure 4:**
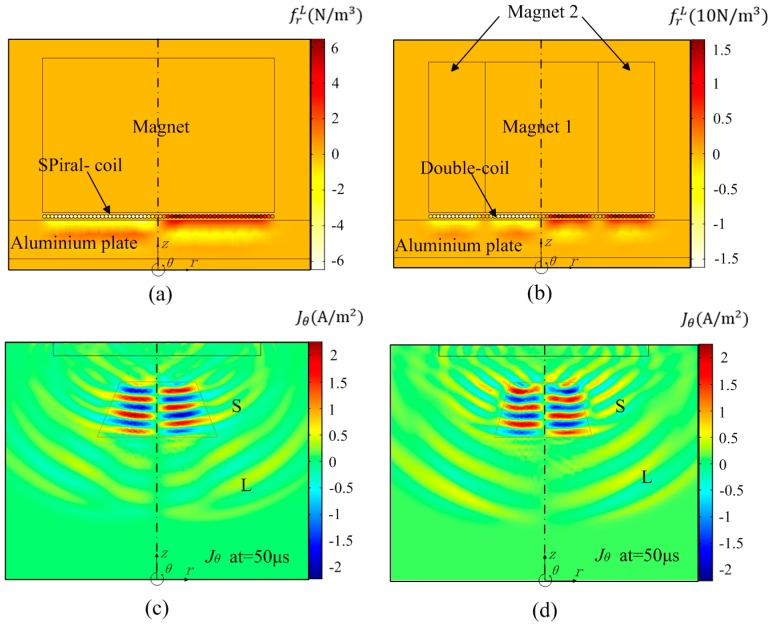
The radial Lorenz force ${f_{r}}^{L}$ produced by (**a**) the conventional spiral-coil EMAT and (**b**) improved one. Pistol graphs of the circumferential eddy density ($J_{\theta }$) generated by (**c**) conventional spiral-coil EMAT and (**d**) improved one at 50 μs.

Figure 4 shows the radial component of Lorenz force $\left({f_{r}}^{L}\right)$ produced by conventional spiral-coil EMAT and improved one, respectively. And the graphs are cross section of 2-D axisymmetric graph rotating around *z* axis. Obviously, the improved double-coil EMAT can generate a consistent circumferential Lorenz force as the conventional one in Figure 4b.

**Figure 5:**
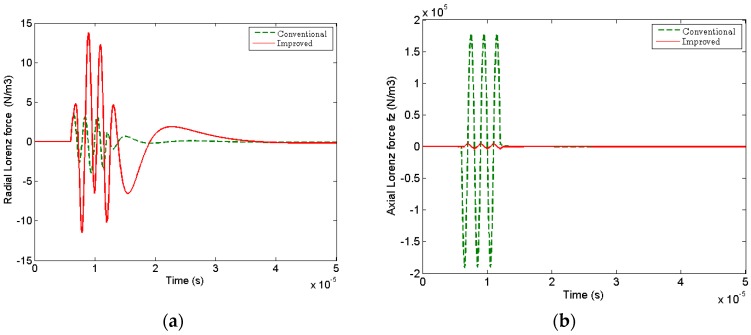
Comparisons between (**a**) radial and (**b**) axial component of Lorenz forces under two configurations, respectively.

However, there is an obvious disparity about the amplitude between radial and axial component of the Lorenz forces for the change of magnet configuration. The radial component of the Lorenz force (${f_{r}}^{L}$) produced by the improved EMAT is dramatically enhanced about 4 times, as shown in Figure 5a. And, the axial component of the Lorenz force (${f_{z}}^{L}$) generated surface waves is greatly weakened 17 times in Figure 5b. This result will be more conducive to the excitation and propagation of bulk waves in metal plate. The area marked by the yellow solid lines in Figure 4c,d shows the snapshots of the circumferential eddy current density ($J_{\theta }$) generated by spiral-coil EMAT and improved one at 50 μs. It is obvious that the eddy current density of the improved double-coil EMAT is more concentrated when the guided waves propagate in the aluminum plate.

**Figure 6:**
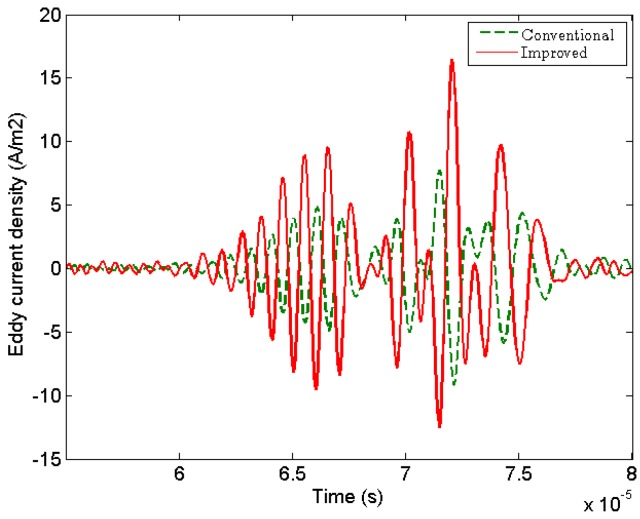
Comparisons of the circumferential eddy current density ($J_{\theta }$) generated by conventional and improved configurations, respectively.

Moreover, in the time domain, the circumferential component of the eddy current density ($J_{\theta }$) measured near the receiving coil is higher than that of the conventional EMAT, as shown in Figure 6. Therefore, the numerical simulation shows that the new configuration is more effective for generating bulk waves.

## 3. Optimization of the Improved Double-Coil EMAT and Experiment Verification

### 3.1. Optimization

**Figure 7:**
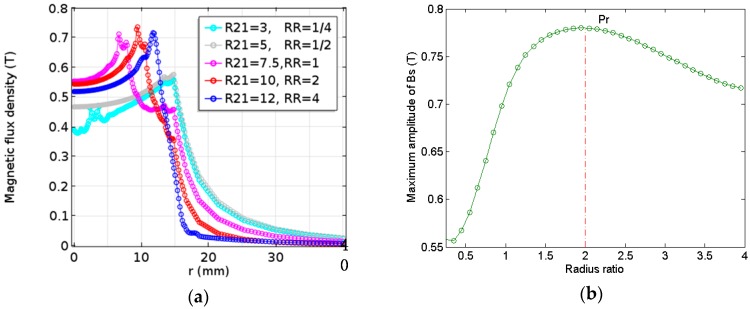
The relationship between the *RR* of the modified magnet and (**a**) the magnetic flux density; and (**b**) the maximum amplitude of the magnetic flux density.

Based on the results of the numerical calculation and analysis of the previous chapter, it is proved that a higher amplitude of the magnetic field density can be obtained by changing the configuration of the magnet. However, the optimum radius ratio ($RR=R_{21}/$($R_{22}$ − $R_{21}$)) of solid magnet (Magnet 1) and toroidal magnet (Magnet 2) of the modified double-coil EMAT is unknown when the maximum magnitude of the magnetic flux density is obtained. To search the optimal result under the improved configuration, the RR and radius of two magnets are optimized. That the RR is divided into five cases to be considered, as shown in Table 2. Here, the overall dimension of the improved magnet is constant ($R_{22}$ = 15 mm, $h_{2}$ = 20 mm). It is very interesting that the influence of the different *RR* on magnetic flux density $\left(B_{s}\right)$ for the improved double-coil EMAT is different, as shown in Figure 7a. Obviously, it can be seen from Figure 7b that, the amplitude of the magnetic flux density increases sharply when the RR increases to the point $P_{r}$, then decreases gradually after it reaches the maximum value. 

When *RR* is roughly equal to 2 ($R_{21}$ = 10, $R_{22}$ = 15) for these cases, there is a maximum magnetic flux density in the improved EMAT. It is well known that the amplitude of the generated ultrasonic wave is proportional to the magnetic flux density [15]. Therefore, the SNR of the new double-coil EMAT can be significantly improved by selecting the optimal *RR*. 

**Figure 8:**
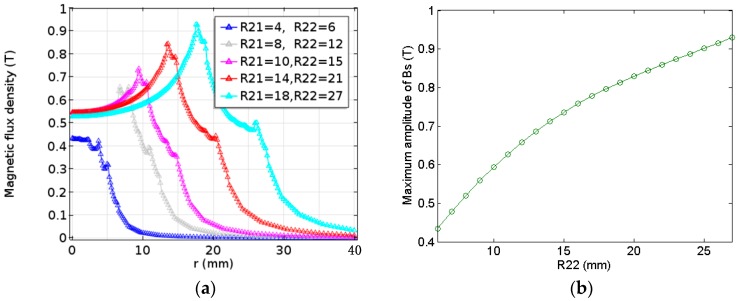
The relationship between the radius of the modified magnet and (**a**) the magnetic flux density; and (**b**) the maximum amplitude of the magnetic flux density.

According to the previous analysis, the magnetic flux density generated under different radius of the improved double-coil EMAT were compared. Here *RR* is set to 2, since the magnitude of the flux density is maximum at this point. As shown in Figure 8a, the magnetic flux density of the modified EMAT tends to grow rapidly with the increase of the radius of the magnet ($R_{22}$) when the radius of the magnet (Magnet 2) changes from 6 mm to 27 mm. It is necessary to be reminded that RR = 2 is maintained continuously. 

**Figure 9:**
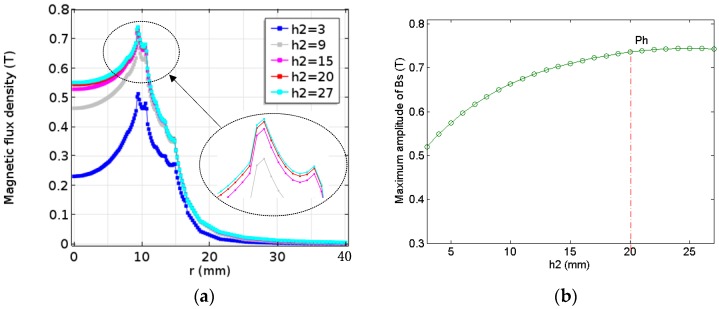
The relationship between the height of the modified magnet and (**a**) the magnetic flux density; and (**b**) the maximum amplitude of the magnetic flux density.

The comparison between the maximum magnetic flux density and the height of the magnet can be seen from Figure 9b that the maximum amplitude increases with increasing the height from the range of 3 to 27 mm. However, the growth rate of the amplitude of the magnetic flux density becomes small after the height increases to point $P_{h}$. As a result, the optimal result with respect to height is approximately 20 mm.

### 3.2. Experiment Analysis

In order to verify the characteristic and performance of the improved double-coil EMAT, several contrastive experiments between the traditional configuration and the improved one are done.

The magnet with radii of 15 mm in the conventional configuration, the solid magnet (Magnet 1) with radii of 10 mm and the ring magnet (Magnet 2) with inner and outer radii of 10 mm and 15 mm in the improved configuration were applied in the experiment. And all the height of the magnet is 20 mm. A double-coil is applied, as shown in Figure 1f, since it is easy to produce a consistent radial Lorenz force with an improved magnet configuration. And the coil dimension of the experiment is the same as that of the previous simulation. The EMAT system is driven by a one-cycle burst signal at 2 MHz to generate and receive bulk waves for measuring the thickness of the aluminum block. All experiments were performed in a pulse-echo mode for thickness measurement.

**Figure 10:**
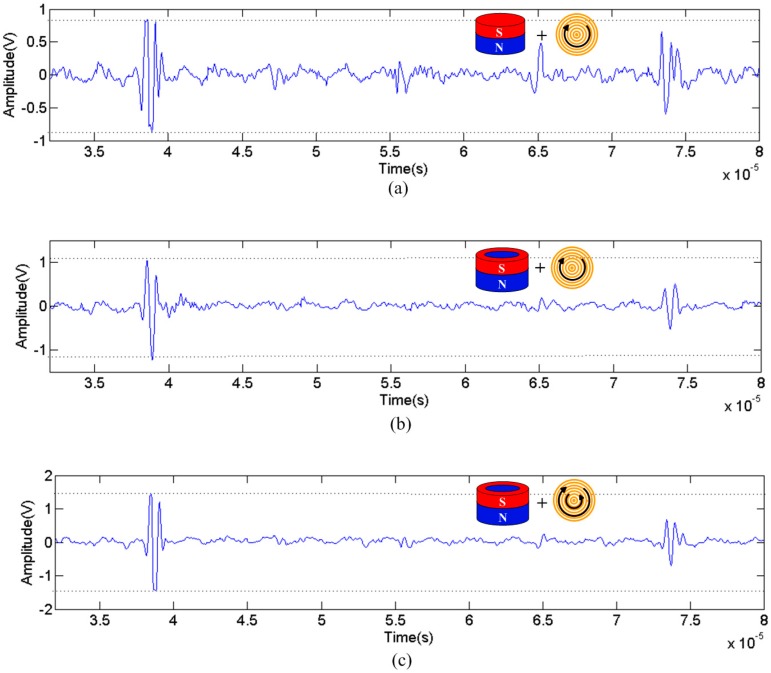
Comparisons of pulse echo signals from the back of the 50-mm-thick aluminum block. (**a**) The conventional configuration; (**b**) The improved configuration without using the double-coil; (**c**) The improved configuration with using the double-coil.

Figure 10 shows the received signals from the aluminum plate with same thickness (50 mm) under the conventional configuration (Figure 10a), the improved magnet configuration without using the double-coil (Figure 10b), and the improved magnet configuration with the double-coil (Figure 10c), respectively. Apparently, it is nearly 38 μs that the first reflected wave from the back wall of the aluminum plate in all experimental arrangements. It is worth noting that the amplitude scale of Figure 10a–c is different.

Compared with Figure 10a, not only the amplitude of the received signal increased by about 25%, but also the noise distortion is reduced significantly in Figure 10b. In the improved configuration, the noise level is similar regardless of whether the coil is changed or not, the amplitude significantly increases when using the double-coil, as shown in Figure 10b,c. Under the traditional configuration, the noise level is ±0.5 V as shown in Figure 10a, the noise amplitude corresponds to approximately 1/5 of the signal generated by the improved configuration, as shown in Figure 10c. Furthermore, under the configuration of Figure 10c, the amplitude peak is the largest in the three cases. According to Equation (1), the logarithm of the SNR ($20\log(V_{\mathrm{EMAT}}/V_{\mathrm{noise}}$)) under three cases were calculated and listed in Table 3. It can be seen that the SNR of the improved configuration with using the double-coil is 20.58 dB, which is 5 times bigger than that of the conventional one.

## 4. Conclusions

This paper presents an improved spiral-coil EMAT for the generation and detection of bulk waves to address the shortcoming of conventional spiral-coil EMAT which is with lower transmission efficiency and higher noise level. Instead of a single cylinder magnet, the improved EMAT configuration consists of an annular magnet (Magnet 2) and a cylinder magnet (Magnet 1) surrounded by the annular one, which are placed oppositely each other in the direction of polarization.

Because the transmission medium of the bulk wave is an aluminum block, 2-D axisymmetric FEM of the two configurations with Lorenz force mechanism were established, respectively. The numerical simulation results show that the maximum amplitude of the magnetic flux density in the surface of the improved magnet is 40% higher than that of the conventional one. And the amplitude of the radial component of Lorenz force generating the bulk wave is increased, but that of the axial component of Lorenz force producing the surface wave is substantially reduced.

Based on the simulation for optimizing the dimension of the improved magnet, it was found that there exists an optimal *RR* which makes the magnetic flux density reach the maximum. When the height and the lift-off distance of the new magnet arrangement is 20 and 0.5 mm, respectively, the *RR* changes from 1/4 to 4, the maximum amplitude of the magnetic flux density increases firstly and then decreases slowly after reaching the peak. Therefore, the optimum RR is found to be approximately 2 as the radius of the cylindrical magnet (Magnet 1) is between 3 and 12 mm.

Furthermore, it can be concluded that improving the arrangement of the magnet and applying the double-coil can effectively improve the low energy conversion efficiency and SNR. According to the experimental results, the improved double-coil EMAT configuration with the combination of Magnet 1 and Magnet 2 yields twice larger amplitude of the pulse-echo signal and less noise disturbance than the conventional one with a single cylinder magnet. It is worth emphasizing that the improved magnet configuration has the function of noise reduction regardless of whether the double-coil is used or not. But it is helpful to improve the amplitude of the pulse-echo signal and conversion efficiency when the double-coil is used.

## Figures and Tables

**Table 1:** Parameters of 2-D FEM used in this paper.

Object	Parameters	Symbol	Value
Spiral-coil	Diameter	$D_{1}$	0.51 mm
Double-coil	Width of the inner coil	$W_{21}$	10 mm
	Width of the outer coil	$W_{22}$	5 mm
	Diameter	$D_{2}$	0.51 mm
Magnet	Radii	$R_{1}$	15 mm
	Height	$h_{1}$	20 mm
Magnet 1	Radii	$R_{21}$	10 mm
	Height	$h_{2}$	20 mm
Magnet 2	Inner radii	$R_{21}$	10 mm
	Outer radii	$R_{22}$	15 mm
	Height	$h_{2}$	20 mm
Al	Mass density	ρ	2700 $\mathrm{kg}/m^{3}$
	Electrical conductivity	σ	3.77 × 10^7^ S/m
	Young’s modulus	E	70 × 10^9^ Pa
	Passion’s ratio	μ	0.33
	Thickness	H	50 mm

**Table 2:** Cases of the *RR* and the variation of the magnetic flux density.

Case	$R_{21}$ (mm)	$R_{22}$ − $R_{21}$ (mm)	$R_{22}$ (mm)	$h_{2}$ (mm)	RR	$B_{s\_max}\left(T\right)$	Increment (%)
1	3	12			1/4	0.568	0.14
2	5	10			1/2	0.576	2.86
3	7.5	7.5	15	20	1	0.712	27.14
4	10	5			2	0.78	39.29
5	12	3			4	0.716	27.86

**Table 3:** Comparisons of SNR in three experiments.

Cases	SNR (dB)
Conventional configuration	4.08
Improved configuration without using double-coil	13.96
Improved configuration with using double-coil	20.58
